# Bis(1-ethyl-4,4′-bipyridin-1-ium) bis­(1,2-di­cyano­ethene-1,2-di­thiol­ato-κ^2^ *S*,*S*′)nickelate(II)

**DOI:** 10.1107/S1600536813015493

**Published:** 2013-07-03

**Authors:** Yao Chen, Wei-hua Ning, Jian-Lan Liu

**Affiliations:** a Department of Applied Chemistry, College of Science, Nanjing University of Technology, Nanjing 210009, People’s Republic of China

## Abstract

In the anion of the title compound, (C_12_H_13_N_2_)[Ni(C_4_N_2_S_2_)_2_], the Ni^II^ atom is coordinated by four S atoms from two 1,2-di­cyano­ethene-1,2-di­thiol­ate (mnt) ligands in a suqare-planar geometry. Weak C—H⋯N and C—H⋯S hydrogen bonds between the 1-ethyl-4,4′-bipyridin-1-ium cations and mnt anions and weak π–π inter­actions between the pyridine rings of the cations [centroid–centroid distances = 3.808 (3) and 3.972 (3) Å] lead to a two-dimensional network parallel to (010).

## Related literature

For general background to bis­(1,2-di­thiol­ene) complexes acting as magnetic materials or showing non-linear optical properties, see: Duan *et al.* (2010 ▶); Kato (2004 ▶). For the synthesis of the compound, see: Pei *et al.* (2010 ▶). For related structures, see: Duan *et al.* (2011 ▶); Liu *et al.* (2011 ▶). 
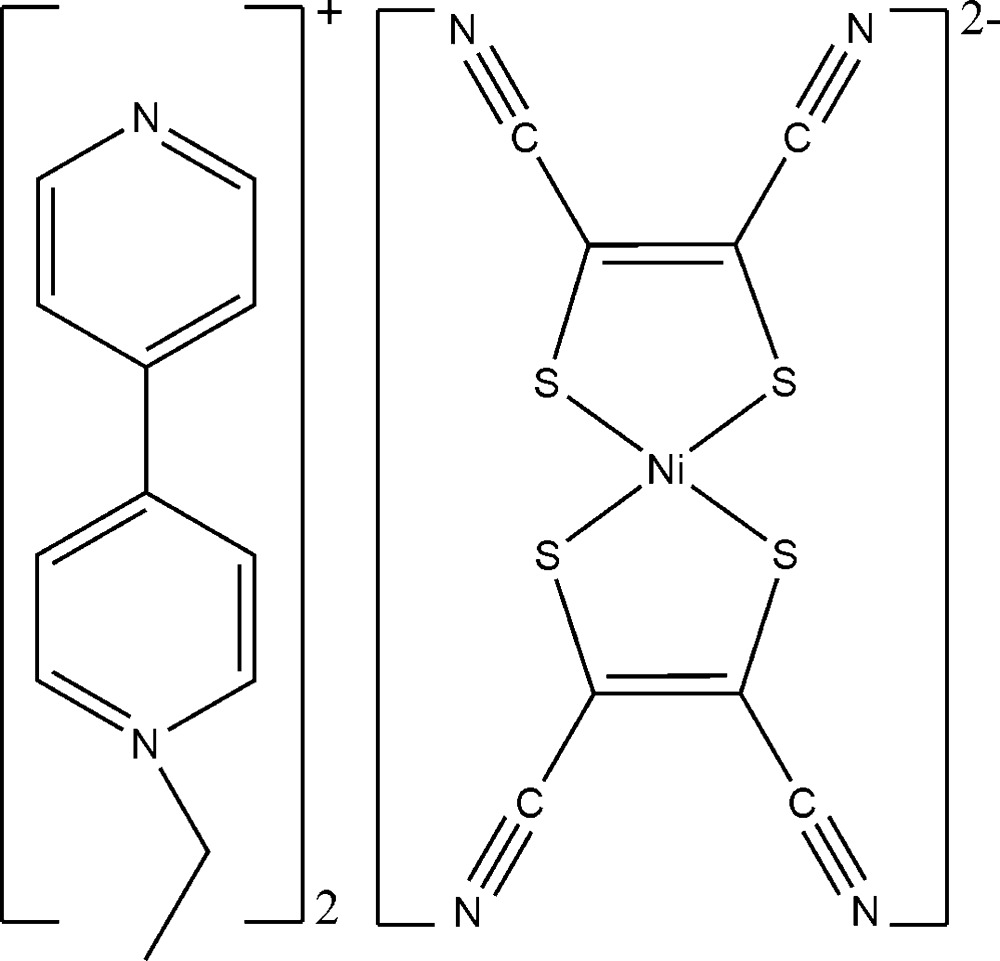


## Experimental

### 

#### Crystal data

(C_12_H_13_N_2_)[Ni(C_4_N_2_S_2_)_2_]
*M* *_r_* = 709.58
Triclinic, 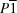

*a* = 7.4505 (13) Å
*b* = 12.793 (2) Å
*c* = 17.745 (3) Å
α = 78.664 (2)°
β = 86.558 (2)°
γ = 80.344 (2)°
*V* = 1634.2 (5) Å^3^
*Z* = 2
Mo *K*α radiation
μ = 0.89 mm^−1^
*T* = 296 K
0.25 × 0.20 × 0.15 mm

#### Data collection

Bruker APEX CCD diffractometer
Absorption correction: multi-scan (*SADABS*; Sheldrick, 1996 ▶) *T*_min_ = 0.808, *T*_max_ = 0.876
14954 measured reflections
7631 independent reflections
3616 reflections with *I* > 2σ(*I*)
*R*_int_ = 0.050

#### Refinement

*R*[*F* ^2^ > 2σ(*F* ^2^)] = 0.051
*wR*(*F* ^2^) = 0.128
*S* = 0.97
7631 reflections
408 parameters
H-atom parameters constrained
Δρ_max_ = 0.49 e Å^−3^
Δρ_min_ = −0.37 e Å^−3^

### 

Data collection: *SMART* (Bruker, 2007 ▶); cell refinement: *SAINT* (Bruker, 2007 ▶); data reduction: *SAINT*; program(s) used to solve structure: *SHELXS97* (Sheldrick, 2008 ▶); program(s) used to refine structure: *SHELXL97* (Sheldrick, 2008 ▶); molecular graphics: *SHELXTL* (Sheldrick, 2008 ▶); software used to prepare material for publication: *SHELXTL*.

## Supplementary Material

Crystal structure: contains datablock(s) D, I. DOI: 10.1107/S1600536813015493/hy2630sup1.cif

Structure factors: contains datablock(s) I. DOI: 10.1107/S1600536813015493/hy2630Isup2.hkl

Additional supplementary materials:  crystallographic information; 3D view; checkCIF report

## Figures and Tables

**Table 1:** Hydrogen-bond geometry (Å, °)

*D*—H⋯*A*	*D*—H	H⋯*A*	*D*⋯*A*	*D*—H⋯*A*
C13—H13⋯S2^i^	0.93	2.78	3.549 (4)	141
C17—H17⋯N4^i^	0.93	2.55	3.441 (6)	161
C21—H21⋯N3^ii^	0.93	2.42	3.342 (6)	170
C22—H22⋯N4^ii^	0.93	2.50	3.372 (5)	156
C24—H24⋯N2	0.93	2.46	3.364 (5)	163
C25—H25⋯N1	0.93	2.52	3.411 (6)	161
C27—H27⋯N4^ii^	0.93	2.58	3.498 (6)	172
C30—H30⋯N2	0.93	2.60	3.526 (6)	176

Symmetry codes: (i) 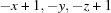; (ii) 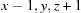.

**Fig. 1.**
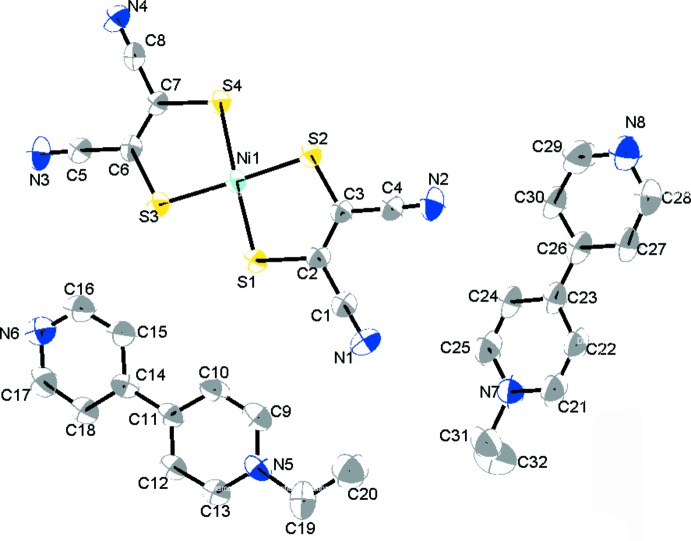
The molecular structure of the title compound. Displacement ellipsoids are drawn at the 30% probability level.

**Fig. 2.**
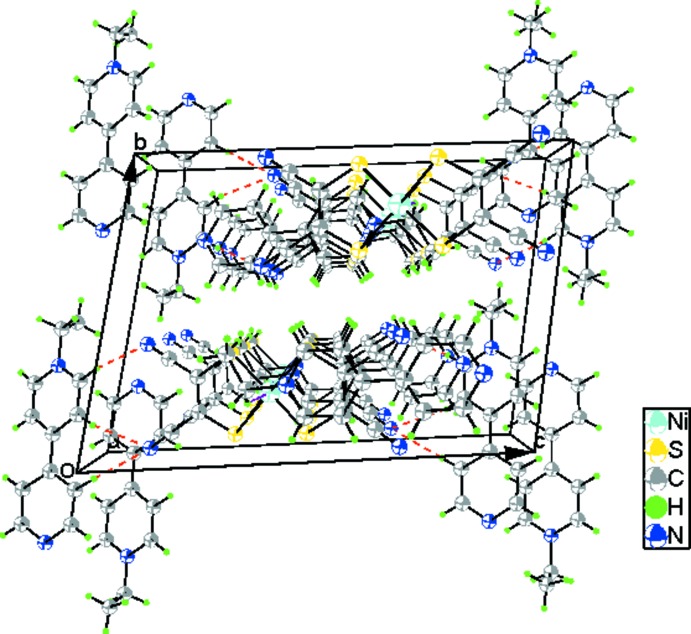
A packing diagram of the title compound viewed down the a axis. Dashed lines indicate hydrogen bonds.

## supplementary crystallographic information

### Comment

Bis(1,2-dithiolene) complexes of transition metals as an important part of the molecular-based materials have been widely studied due to their novel applications in the areas of materials science, medicines and biology (Duan *et al.*, 2010; Kato, 2004). It is known that weak inter- or intramolecular interactions in the complexes could influence on their properties. Herein we report the crystal structure of the title compound. The bond lengths and angles in the title compound (Fig. 1) are within normal ranges (Duan *et al.*, 2011; Liu *et al.*, 2011). Weak C—H···N and C—H···S hydrogen bonds between the 1-ethyl-4,4'-bipyridin-1-ium cations and 1,2-dicyanoethene-1,2-dithiolate anions and weak π–π interactions between the pyridine rings of the cations [centroid–centroid distances = 3.808 (3) and 3.972 (3) Å] lead to a two-dimensional network parallel to (010) (Fig. 2).

### Experimental

The title compound was prepared by a method similar to that reported in literature (Pei *et al.*, 2010). Nickel chloride hexahydrate (238 mg, 1.00 mmol) and disodium maleonitriledithiolate (365 mg, 2.00 mmol) were mixed under stirring in water (50 ml) and heated to boiling for about 20 min. After filtering the red solution, an aequeous solution of 1-ethyl-4,4'-bipyridin-1-ium bromide (554 mg, 2.00 mmol) was added dropwise to the filtrate. The immediately formed dark red precipitate was filtered off, washed with water and dried in vacuum oven, giving the crude product (yield: 511 mg, 72%). Red block-like single crystals were obtained by slow evaporation of the crude in an acetonitrile solution at room temperature in about two weeks.

### Refinement

H atoms were positioned geometrically and refined as riding atoms, with C—H = 0.93 (CH), 0.97 (CH_2_) and 0.96 (CH_3_) Å and with *U*_iso_(H) = 1.2(1.5 for methyl)*U*_eq_(C).

### Crystal data

**Table:**

(C_12_H_13_N_2_)[Ni(C_4_N_2_S_2_)_2_]	*Z* = 2
*M**_r_* = 709.58	*F*(000) = 732.0
Triclinic, *P*1	*D*_x_ = 1.442 Mg m^−^^3^
Hall symbol: -P 1	Mo *K*α radiation, λ = 0.71073 Å
*a* = 7.4505 (13) Å	Cell parameters from 1518 reflections
*b* = 12.793 (2) Å	θ = 2.3–19.7°
*c* = 17.745 (3) Å	µ = 0.89 mm^−^^1^
α = 78.664 (2)°	*T* = 296 K
β = 86.558 (2)°	Block, red
γ = 80.344 (2)°	0.25 × 0.20 × 0.15 mm
*V* = 1634.2 (5) Å^3^	

### Data collection

**Table:**

Bruker APEX CCD diffractometer	7631 independent reflections
Radiation source: fine-focus sealed tube	3616 reflections with *I* > 2σ(*I*)
Graphite monochromator	*R*_int_ = 0.050
φ and ω scans	θ_max_ = 28.0°, θ_min_ = 1.6°
Absorption correction: multi-scan (*SADABS*; Sheldrick, 1996)	*h* = −9→9
*T*_min_ = 0.808, *T*_max_ = 0.876	*k* = −16→16
14954 measured reflections	*l* = −23→20

### Refinement

**Table:**

Refinement on *F*^2^	Primary atom site location: structure-invariant direct methods
Least-squares matrix: full	Secondary atom site location: difference Fourier map
*R*[*F*^2^ > 2σ(*F*^2^)] = 0.051	Hydrogen site location: inferred from neighbouring sites
*wR*(*F*^2^) = 0.128	H-atom parameters constrained
*S* = 0.97	*w* = 1/[σ^2^(*F*_o_^2^) + (0.0418*P*)^2^] where *P* = (*F*_o_^2^ + 2*F*_c_^2^)/3
7631 reflections	(Δ/σ)_max_ < 0.001
408 parameters	Δρ_max_ = 0.49 e Å^−^^3^
0 restraints	Δρ_min_ = −0.37 e Å^−^^3^

### Special details

**Table:**

Geometry. All esds (except the esd in the dihedral angle between two l.s. planes) are estimated using the full covariance matrix. The cell esds are taken into account individually in the estimation of esds in distances, angles and torsion angles; correlations between esds in cell parameters are only used when they are defined by crystal symmetry. An approximate (isotropic) treatment of cell esds is used for estimating esds involving l.s. planes.
Refinement. Refinement of F^2^ against ALL reflections. The weighted R-factor wR and goodness of fit S are based on F^2^, conventional R-factors R are based on F, with F set to zero for negative F^2^. The threshold expression of F^2^ > 2sigma(F^2^) is used only for calculating R-factors(gt) etc. and is not relevant to the choice of reflections for refinement. R-factors based on F^2^ are statistically about twice as large as those based on F, and R- factors based on ALL data will be even larger.

### Fractional atomic coordinates and isotropic or equivalent isotropic displacement parameters (Å^2^)

**Table:**

	*x*	*y*	*z*	*U*_iso_*/*U*_eq_	
Ni1	0.82715 (7)	0.22869 (4)	0.39454 (3)	0.04134 (16)	
S1	0.73997 (14)	0.35458 (7)	0.46177 (6)	0.0484 (3)	
S2	0.76184 (16)	0.10350 (7)	0.48848 (6)	0.0547 (3)	
S3	0.87246 (15)	0.35324 (8)	0.29580 (6)	0.0528 (3)	
S4	0.93428 (17)	0.10088 (8)	0.33202 (6)	0.0613 (3)	
C2	0.6768 (5)	0.2822 (3)	0.5502 (2)	0.0441 (9)	
C1	0.6135 (6)	0.3411 (3)	0.6099 (2)	0.0499 (10)	
C3	0.6833 (5)	0.1733 (3)	0.5608 (2)	0.0440 (10)	
C4	0.6232 (6)	0.1130 (3)	0.6311 (2)	0.0551 (11)	
C6	0.9759 (5)	0.2797 (3)	0.2284 (2)	0.0465 (10)	
C5	1.0367 (6)	0.3382 (3)	0.1563 (2)	0.0565 (12)	
C7	1.0008 (5)	0.1697 (3)	0.2436 (2)	0.0479 (10)	
C8	1.0813 (6)	0.1076 (3)	0.1876 (2)	0.0558 (11)	
C9	0.2604 (6)	0.3605 (3)	0.4593 (3)	0.0606 (12)	
H9	0.2870	0.4231	0.4273	0.073*	
C10	0.1955 (5)	0.3644 (3)	0.5330 (3)	0.0576 (12)	
H10	0.1772	0.4301	0.5497	0.069*	
C11	0.1563 (5)	0.2723 (3)	0.5835 (2)	0.0461 (10)	
C12	0.1837 (6)	0.1787 (3)	0.5540 (2)	0.0577 (12)	
H12	0.1594	0.1148	0.5850	0.069*	
C13	0.2461 (6)	0.1784 (3)	0.4801 (3)	0.0609 (12)	
H13	0.2615	0.1140	0.4618	0.073*	
C14	0.0815 (5)	0.2768 (3)	0.6632 (2)	0.0481 (10)	
C15	0.0630 (6)	0.3698 (3)	0.6932 (3)	0.0672 (13)	
H15	0.1012	0.4316	0.6645	0.081*	
C16	−0.0124 (6)	0.3709 (4)	0.7661 (3)	0.0713 (14)	
H16	−0.0215	0.4345	0.7849	0.086*	
C17	−0.0516 (7)	0.1977 (4)	0.7814 (3)	0.0785 (15)	
H17	−0.0891	0.1368	0.8117	0.094*	
C18	0.0220 (6)	0.1890 (3)	0.7087 (3)	0.0645 (13)	
H18	0.0309	0.1244	0.6911	0.077*	
C19	0.3576 (6)	0.2552 (4)	0.3530 (2)	0.0682 (13)	
H19A	0.4785	0.2126	0.3566	0.082*	
H19B	0.2795	0.2159	0.3312	0.082*	
C20	0.3667 (7)	0.3600 (4)	0.3003 (3)	0.0956 (17)	
H20A	0.2507	0.4057	0.3006	0.143*	
H20B	0.3972	0.3476	0.2491	0.143*	
H20C	0.4582	0.3943	0.3170	0.143*	
C21	0.2644 (6)	0.2746 (4)	0.9515 (3)	0.0754 (14)	
H21	0.2103	0.3136	0.9883	0.090*	
C22	0.2539 (6)	0.1671 (4)	0.9611 (2)	0.0686 (13)	
H22	0.1919	0.1345	1.0040	0.082*	
C23	0.3346 (5)	0.1053 (4)	0.9076 (2)	0.0567 (11)	
C24	0.4248 (6)	0.1605 (4)	0.8456 (2)	0.0688 (13)	
H24	0.4823	0.1231	0.8085	0.083*	
C25	0.4311 (7)	0.2679 (4)	0.8376 (3)	0.0796 (15)	
H25	0.4917	0.3027	0.7951	0.095*	
C26	0.3254 (6)	−0.0126 (4)	0.9179 (2)	0.0572 (11)	
C27	0.2592 (6)	−0.0700 (4)	0.9857 (3)	0.0676 (13)	
H27	0.2166	−0.0359	1.0264	0.081*	
C28	0.2581 (6)	−0.1785 (4)	0.9909 (3)	0.0759 (14)	
H28	0.2132	−0.2155	1.0367	0.091*	
C29	0.3719 (8)	−0.1787 (5)	0.8721 (3)	0.0989 (19)	
H29	0.4079	−0.2151	0.8318	0.119*	
C30	0.3822 (7)	−0.0688 (4)	0.8589 (3)	0.0785 (15)	
H30	0.4259	−0.0341	0.8121	0.094*	
C31	0.3614 (8)	0.4438 (4)	0.8812 (3)	0.0931 (17)	
H31A	0.3367	0.4788	0.8284	0.112*	
H31B	0.2689	0.4765	0.9140	0.112*	
C32	0.5411 (9)	0.4616 (4)	0.9009 (4)	0.120 (2)	
H32A	0.5715	0.4209	0.9512	0.181*	
H32B	0.5383	0.5370	0.9005	0.181*	
H32C	0.6308	0.4386	0.8640	0.181*	
N1	0.5638 (5)	0.3873 (3)	0.6585 (2)	0.0711 (11)	
N2	0.5710 (6)	0.0624 (3)	0.6863 (2)	0.0819 (13)	
N3	1.0878 (6)	0.3863 (3)	0.1002 (2)	0.0855 (13)	
N4	1.1447 (6)	0.0573 (3)	0.1433 (2)	0.0788 (13)	
N5	0.2860 (4)	0.2673 (3)	0.4330 (2)	0.0513 (9)	
N6	−0.0723 (5)	0.2883 (3)	0.8109 (2)	0.0716 (11)	
N7	0.3506 (5)	0.3244 (3)	0.8906 (2)	0.0708 (11)	
N8	0.3154 (6)	−0.2354 (4)	0.9367 (3)	0.0889 (13)	

### Atomic displacement parameters (Å^2^)

**Table:**

	*U*^11^	*U*^22^	*U*^33^	*U*^12^	*U*^13^	*U*^23^
Ni1	0.0509 (3)	0.0353 (3)	0.0370 (3)	−0.0089 (2)	0.0064 (2)	−0.0055 (2)
S1	0.0639 (7)	0.0369 (5)	0.0446 (6)	−0.0108 (5)	0.0060 (5)	−0.0085 (4)
S2	0.0809 (8)	0.0356 (6)	0.0452 (7)	−0.0104 (5)	0.0182 (6)	−0.0072 (5)
S3	0.0705 (8)	0.0391 (6)	0.0450 (7)	−0.0087 (5)	0.0151 (6)	−0.0043 (5)
S4	0.0992 (10)	0.0382 (6)	0.0447 (7)	−0.0145 (6)	0.0226 (6)	−0.0081 (5)
C2	0.045 (2)	0.046 (2)	0.041 (2)	−0.0056 (18)	0.0023 (19)	−0.0091 (18)
C1	0.055 (3)	0.049 (2)	0.045 (3)	−0.006 (2)	0.001 (2)	−0.009 (2)
C3	0.051 (3)	0.041 (2)	0.037 (2)	−0.0031 (19)	0.0066 (19)	−0.0056 (18)
C4	0.069 (3)	0.048 (2)	0.045 (3)	−0.005 (2)	0.014 (2)	−0.009 (2)
C6	0.054 (3)	0.049 (2)	0.034 (2)	−0.009 (2)	0.003 (2)	−0.0032 (18)
C5	0.077 (3)	0.048 (3)	0.043 (3)	−0.008 (2)	0.010 (2)	−0.010 (2)
C7	0.065 (3)	0.044 (2)	0.036 (2)	−0.010 (2)	0.009 (2)	−0.0108 (18)
C8	0.079 (3)	0.050 (3)	0.035 (3)	−0.012 (2)	0.002 (2)	0.000 (2)
C9	0.064 (3)	0.047 (3)	0.070 (3)	−0.015 (2)	−0.001 (3)	−0.004 (2)
C10	0.066 (3)	0.035 (2)	0.074 (3)	−0.014 (2)	0.003 (3)	−0.011 (2)
C11	0.047 (2)	0.035 (2)	0.057 (3)	−0.0102 (18)	−0.007 (2)	−0.0064 (19)
C12	0.075 (3)	0.040 (2)	0.059 (3)	−0.015 (2)	0.002 (3)	−0.007 (2)
C13	0.077 (3)	0.046 (3)	0.061 (3)	−0.012 (2)	0.002 (3)	−0.011 (2)
C14	0.045 (2)	0.041 (2)	0.058 (3)	−0.0047 (19)	0.000 (2)	−0.011 (2)
C15	0.086 (4)	0.048 (3)	0.070 (3)	−0.014 (2)	0.012 (3)	−0.018 (2)
C16	0.089 (4)	0.054 (3)	0.075 (4)	−0.016 (3)	0.004 (3)	−0.021 (3)
C17	0.101 (4)	0.057 (3)	0.076 (4)	−0.019 (3)	0.014 (3)	−0.008 (3)
C18	0.083 (3)	0.046 (3)	0.064 (3)	−0.013 (2)	0.003 (3)	−0.009 (2)
C19	0.056 (3)	0.092 (4)	0.053 (3)	−0.020 (3)	−0.005 (2)	0.005 (3)
C20	0.098 (4)	0.098 (4)	0.093 (4)	−0.020 (3)	0.003 (3)	−0.021 (4)
C21	0.083 (4)	0.089 (4)	0.047 (3)	−0.003 (3)	0.016 (3)	−0.010 (3)
C22	0.078 (3)	0.080 (3)	0.041 (3)	−0.007 (3)	0.017 (2)	−0.007 (2)
C23	0.049 (3)	0.075 (3)	0.042 (3)	−0.002 (2)	0.002 (2)	−0.009 (2)
C24	0.086 (4)	0.077 (3)	0.043 (3)	−0.016 (3)	0.017 (3)	−0.012 (2)
C25	0.090 (4)	0.088 (4)	0.054 (3)	−0.013 (3)	0.020 (3)	−0.007 (3)
C26	0.049 (3)	0.081 (3)	0.041 (3)	−0.010 (2)	0.003 (2)	−0.013 (2)
C27	0.066 (3)	0.085 (4)	0.049 (3)	−0.014 (3)	0.012 (2)	−0.008 (3)
C28	0.071 (4)	0.090 (4)	0.064 (4)	−0.018 (3)	0.001 (3)	−0.007 (3)
C29	0.121 (5)	0.095 (4)	0.086 (4)	−0.027 (4)	0.033 (4)	−0.031 (4)
C30	0.100 (4)	0.082 (4)	0.053 (3)	−0.017 (3)	0.022 (3)	−0.017 (3)
C31	0.109 (5)	0.070 (4)	0.084 (4)	0.010 (3)	−0.003 (4)	0.005 (3)
C32	0.144 (6)	0.083 (4)	0.137 (6)	−0.017 (4)	−0.003 (5)	−0.026 (4)
N1	0.094 (3)	0.067 (3)	0.054 (3)	−0.006 (2)	0.009 (2)	−0.026 (2)
N2	0.112 (3)	0.075 (3)	0.051 (3)	−0.016 (2)	0.025 (2)	−0.001 (2)
N3	0.124 (4)	0.074 (3)	0.053 (3)	−0.024 (3)	0.029 (3)	−0.001 (2)
N4	0.123 (4)	0.062 (3)	0.052 (3)	−0.014 (2)	0.023 (2)	−0.021 (2)
N5	0.046 (2)	0.047 (2)	0.060 (2)	−0.0098 (17)	−0.0047 (18)	−0.0043 (18)
N6	0.088 (3)	0.062 (3)	0.068 (3)	−0.011 (2)	0.006 (2)	−0.020 (2)
N7	0.078 (3)	0.073 (3)	0.052 (3)	0.001 (2)	0.005 (2)	−0.003 (2)
N8	0.101 (4)	0.087 (3)	0.080 (3)	−0.022 (3)	0.013 (3)	−0.018 (3)

### Geometric parameters (Å, º)

**Table:**

Ni1—S1	2.1796 (11)	C17—H17	0.9300
Ni1—S2	2.1668 (11)	C18—H18	0.9300
Ni1—S4	2.1703 (11)	C19—C20	1.485 (6)
Ni1—S3	2.1760 (11)	C19—N5	1.515 (5)
S1—C2	1.740 (4)	C19—H19A	0.9700
S2—C3	1.723 (4)	C19—H19B	0.9700
S3—C6	1.731 (4)	C20—H20A	0.9600
S4—C7	1.730 (4)	C20—H20B	0.9600
C2—C3	1.362 (5)	C20—H20C	0.9600
C2—C1	1.431 (5)	C21—N7	1.331 (5)
C1—N1	1.149 (4)	C21—C22	1.367 (6)
C3—C4	1.422 (5)	C21—H21	0.9300
C4—N2	1.148 (5)	C22—C23	1.398 (5)
C6—C7	1.362 (5)	C22—H22	0.9300
C6—C5	1.440 (5)	C23—C24	1.388 (5)
C5—N3	1.143 (5)	C23—C26	1.496 (6)
C7—C8	1.434 (5)	C24—C25	1.362 (6)
C8—N4	1.145 (5)	C24—H24	0.9300
C9—N5	1.346 (5)	C25—N7	1.350 (5)
C9—C10	1.374 (5)	C25—H25	0.9300
C9—H9	0.9300	C26—C27	1.390 (5)
C10—C11	1.395 (5)	C26—C30	1.390 (5)
C10—H10	0.9300	C27—C28	1.374 (6)
C11—C12	1.378 (5)	C27—H27	0.9300
C11—C14	1.498 (5)	C28—N8	1.326 (5)
C12—C13	1.366 (5)	C28—H28	0.9300
C12—H12	0.9300	C29—N8	1.317 (6)
C13—N5	1.338 (5)	C29—C30	1.394 (6)
C13—H13	0.9300	C29—H29	0.9300
C14—C18	1.370 (5)	C30—H30	0.9300
C14—C15	1.379 (5)	C31—C32	1.468 (7)
C15—C16	1.380 (6)	C31—N7	1.519 (6)
C15—H15	0.9300	C31—H31A	0.9700
C16—N6	1.316 (5)	C31—H31B	0.9700
C16—H16	0.9300	C32—H32A	0.9600
C17—N6	1.346 (5)	C32—H32B	0.9600
C17—C18	1.388 (6)	C32—H32C	0.9600
			
S2—Ni1—S4	87.25 (4)	C20—C19—H19B	108.9
S2—Ni1—S3	175.30 (5)	N5—C19—H19B	108.9
S4—Ni1—S3	91.97 (4)	H19A—C19—H19B	107.7
S2—Ni1—S1	92.18 (4)	C19—C20—H20A	109.5
S4—Ni1—S1	175.51 (5)	C19—C20—H20B	109.5
S3—Ni1—S1	88.96 (4)	H20A—C20—H20B	109.5
C2—S1—Ni1	102.87 (13)	C19—C20—H20C	109.5
C3—S2—Ni1	103.49 (13)	H20A—C20—H20C	109.5
C6—S3—Ni1	103.08 (13)	H20B—C20—H20C	109.5
C7—S4—Ni1	103.58 (13)	N7—C21—C22	121.2 (4)
C3—C2—C1	121.6 (3)	N7—C21—H21	119.4
C3—C2—S1	120.4 (3)	C22—C21—H21	119.4
C1—C2—S1	117.9 (3)	C21—C22—C23	121.3 (4)
N1—C1—C2	179.2 (5)	C21—C22—H22	119.4
C2—C3—C4	121.5 (3)	C23—C22—H22	119.4
C2—C3—S2	121.0 (3)	C24—C23—C22	115.5 (4)
C4—C3—S2	117.5 (3)	C24—C23—C26	122.8 (4)
N2—C4—C3	177.3 (5)	C22—C23—C26	121.7 (4)
C7—C6—C5	121.0 (3)	C25—C24—C23	121.6 (4)
C7—C6—S3	121.0 (3)	C25—C24—H24	119.2
C5—C6—S3	118.0 (3)	C23—C24—H24	119.2
N3—C5—C6	177.9 (5)	N7—C25—C24	120.8 (5)
C6—C7—C8	121.7 (3)	N7—C25—H25	119.6
C6—C7—S4	120.3 (3)	C24—C25—H25	119.6
C8—C7—S4	118.0 (3)	C27—C26—C30	117.4 (4)
N4—C8—C7	179.4 (5)	C27—C26—C23	122.0 (4)
N5—C9—C10	120.8 (4)	C30—C26—C23	120.6 (4)
N5—C9—H9	119.6	C28—C27—C26	118.4 (5)
C10—C9—H9	119.6	C28—C27—H27	120.8
C9—C10—C11	121.6 (4)	C26—C27—H27	120.8
C9—C10—H10	119.2	N8—C28—C27	125.9 (5)
C11—C10—H10	119.2	N8—C28—H28	117.0
C12—C11—C10	115.7 (4)	C27—C28—H28	117.0
C12—C11—C14	122.7 (3)	N8—C29—C30	125.5 (5)
C10—C11—C14	121.6 (3)	N8—C29—H29	117.3
C13—C12—C11	121.0 (4)	C30—C29—H29	117.3
C13—C12—H12	119.5	C26—C30—C29	118.0 (5)
C11—C12—H12	119.5	C26—C30—H30	121.0
N5—C13—C12	122.4 (4)	C29—C30—H30	121.0
N5—C13—H13	118.8	C32—C31—N7	111.8 (4)
C12—C13—H13	118.8	C32—C31—H31A	109.3
C18—C14—C15	117.0 (4)	N7—C31—H31A	109.3
C18—C14—C11	120.9 (4)	C32—C31—H31B	109.3
C15—C14—C11	122.0 (4)	N7—C31—H31B	109.3
C14—C15—C16	119.7 (4)	H31A—C31—H31B	107.9
C14—C15—H15	120.1	C31—C32—H32A	109.5
C16—C15—H15	120.1	C31—C32—H32B	109.5
N6—C16—C15	124.8 (4)	H32A—C32—H32B	109.5
N6—C16—H16	117.6	C31—C32—H32C	109.5
C15—C16—H16	117.6	H32A—C32—H32C	109.5
N6—C17—C18	124.4 (4)	H32B—C32—H32C	109.5
N6—C17—H17	117.8	C13—N5—C9	118.5 (4)
C18—C17—H17	117.8	C13—N5—C19	117.0 (3)
C14—C18—C17	119.1 (4)	C9—N5—C19	124.5 (4)
C14—C18—H18	120.5	C16—N6—C17	114.9 (4)
C17—C18—H18	120.5	C21—N7—C25	119.6 (4)
C20—C19—N5	113.4 (4)	C21—N7—C31	120.4 (4)
C20—C19—H19A	108.9	C25—N7—C31	120.0 (4)
N5—C19—H19A	108.9	C29—N8—C28	114.7 (5)

### Hydrogen-bond geometry (Å, º)

**Table:**

*D*—H···*A*	*D*—H	H···*A*	*D*···*A*	*D*—H···*A*
C13—H13···S2^i^	0.93	2.78	3.549 (4)	141
C17—H17···N4^i^	0.93	2.55	3.441 (6)	161
C21—H21···N3^ii^	0.93	2.42	3.342 (6)	170
C22—H22···N4^ii^	0.93	2.50	3.372 (5)	156
C24—H24···N2	0.93	2.46	3.364 (5)	163
C25—H25···N1	0.93	2.52	3.411 (6)	161
C27—H27···N4^ii^	0.93	2.58	3.498 (6)	172
C30—H30···N2	0.93	2.60	3.526 (6)	176

Symmetry codes: (i) −*x*+1, −*y*, −*z*+1; (ii) *x*−1, *y*, *z*+1.
